# Flavonoids as Strong Inhibitors of MAPK3: A Computational Drug Discovery Approach

**DOI:** 10.1155/2023/8899240

**Published:** 2023-04-14

**Authors:** Amir Taherkhani, Parita Khodadadi, Lida Samie, Zahra Azadian, Zeynab Bayat

**Affiliations:** ^1^Research Center for Molecular Medicine, Hamadan University of Medical Sciences, Hamadan, Iran; ^2^Department of Oral and Maxillofacial Medicine, Faculty of Dentistry, Hamadan University of Medical Sciences, Hamadan, Iran

## Abstract

**Background:**

Mitogen-activated protein kinase 3 (MAPK3) mediates the onset, progression, metastasis, drug resistance, and poor prognosis in various malignancies, including glioma, liver, ovarian, thyroid, lung, breast, gastric, and oral cancers. Negative regulation of MAPK3 expression using miRNAs has led to therapeutic effects in cancer.

**Objectives:**

The present study performed molecular docking and dynamics simulation to identify potential MAPK3 inhibitors from natural flavonoids, possibly leading to drug development in cancer therapy.

**Methods:**

A computational drug discovery approach was performed using the AutoDock tool to identify potential MAPK3 inhibitors from 46 plant-based flavonoids. A cross-validation study was executed using the Schrödinger Maestro docking tool. Molecular dynamics (MD) was executed to evaluate the stability of docked poses between the top-ranked compounds and the MAPK3 catalytic domain. Interactions among the most potent MAPK3 inhibitors and residues within the receptor's active site were studied using the BIOVIA Discovery Studio Visualizer before and after 100 ns MD simulations.

**Results:**

Kaempferol 3-rutinoside-4′-glucoside, kaempferol 3-rutinoside-7-sophoroside, rutin, and vicenin-2 exhibited a magnificent binding affinity to the receptor's active site. In addition, the stability of the docked poses of these compounds seemed to be stable after ∼45 ns computer simulations.

**Conclusion:**

The present study suggests that kaempferol 3-rutinoside-4′-glucoside, kaempferol 3-rutinoside-7-sophoroside, rutin, and vicenin-2 could strongly bind to the MAPK3 catalytic site and could be assigned as a potent inhibitor for MAPK3. These findings may be helpful in the treatment of various cancers. However, further validation experiments are needed.

## 1. Introduction

Mitogen-activated protein kinase 3 (MAPK3), called extracellular signal-regulated kinase 1 (ERK1), is a critical ERK/MAPK pathway cell signaling molecule. It mediates the transmission of signals from a cell's exterior to its interior. The ERK/MAPK pathway regulates apoptosis, cell proliferation, and migration [1, 2]. MAPK3 phosphorylates its downstream cytoplasmic protein, activating several nuclear transcription factors (e.g., c-Jun and c-fos) and participating in apoptosis and cell proliferation [3, 4]. The overexpression and/or hyperactivity of MAPK3 has been linked to the initiation, development, cancer cell migration, and drug resistance in various carcinomas, including liver, thyroid, lung, and gastric cancers [5]. Previously, Du et al. [6] reported that miR-143 upregulation reduced breast cancer cell proliferation by targeting MAPK3. Additionally, Cao et al. [5] demonstrated that the reduced miR-129 expression and MAPK3 overexpression were associated with cisplatin resistance in gastric cancer cells. MiR-129 overexpression also diminished the cell proliferation via downregulating MAPK3 leading to enhanced cell apoptosis. These results confirm the significant role of MAPK3 in tumorigenesis and drug resistance in different cancers.

Molecular docking analysis is a structural bioinformatics approach commonly used for drug discovery, identifying potential inhibitors for a given biological target. Molecular docking also unravels interaction modes between macromolecules and drug candidates [7, 8]. Molecular docking analysis includes three steps: macromolecule structure, preparation of small molecule structure, and evaluation of binding affinity between ligand and receptor [9]. Postdocking studies such as molecular dynamics (MD) analysis are frequently used to understand better ligands' dynamic manner and stability within the receptors' binding site (e.g., proteins' active site). Moreover, MD enables assessing the flexibility of residues inside the catalytic site of proteins [10–12].

Flavonoids are secondary metabolites predominantly found in the plant kingdom, playing a significant role in plant development [13]. The basic structure of flavonoids has been shown in our previous study [14]. Generally, they comprise a C6-C3-C6 skeleton, in which the C6 rings are aromatic, and the C3 is a bridge linker [15]. Due to their high binding affinity to enzymes [16], flavonoids have demonstrated a wide range of pharmacological behaviors in medicine, including anticancer, antioxidant, anti-inflammatory, antiallergic, antibacterial, and antiviral properties [17, 18]. There is growing evidence suggesting the curative potential of flavonoids in different cancers such as breast [19], colorectal [20], oral [21], and lung cancers [22], as well as hepatocellular carcinoma [19].

The present study suggested that flavonoids may act as potential inhibitors of MAPK3 activity, leading to downregulating its downstream signaling pathways and reducing cell proliferation and migration. Therefore, we performed a molecular docking analysis to evaluate the binding affinity of several flavonoids to the MAPK3 active site. According to estimated inhibition constant values (*K*i) between studied ligands and the protein active site, top-ranked MAPK3 inhibitors were introduced, interaction modes among top-ranked flavonoids and residues inside the MAPK3 catalytic site were analyzed, and the stability of the docked pose of the best MAPK3 inhibitor was studied by executing MD simulation. The present results might be beneficial in cancer treatment.

## 2. Materials and Methods

### 2.1. Structural Preparation of MAPK3 and Flavonoids

The three-dimensional structure of MAPK3 was downloaded at 1.4 Å X-ray resolution from the RCSB database [23], which is available at https://www.rcsb.org (PDB ID: 4QTB) [24]. The 4QTB file included two polypeptide chains: A and B. The total number of residues in each chain was checked using the Notepad++ tool. Accordingly, chains A and B included 351 and 348 residues, respectively. Therefore, chain A was selected for further analysis. Critical amino acids within the active site were identified by analyzing the two structure interactions among the 38Z (positive control inhibitor of the protein with a PDB ID of 24866313) and residues inside the active site of the protein using the BIOVIA Discovery Studio Visualizer version 19.1.0.18287, as well as reviewing the study by Chaikuad et al. [25]. Next, the 38Z molecule was eliminated from the PDB file, and energy optimization was executed via the Swiss-pdbViewer version 4.1.0, available at https://spdbv.unil.ch [26].

Previous studies have reported about 6000 flavonoids contributing to the colorful pigments of fruits, vegetables, and medicinal herbs [27]. The present study selected 46 flavonoids mainly found in commonly used fruits and vegetables, including onions, lettuce, kale, apples, tomatoes, berries, grapes, red grapes, red grapes, raspberries, strawberries, bilberries, merlot grapes, blueberries, and blackberries [27]. Therefore, 46 natural flavonoids were considered for identifying possible MAPK3 inhibitors, and the binding affinity of a standard drug (name: Purvalanol; PubChem ID: 448991; DrugBank ID: DB02733) to the active site of MAPK3 was regarded as a positive control inhibitor in this study. Structural preparation and energy minimization of flavonoids were explained in our previous study [28].

### 2.2. Molecular Docking

AutoDock 4.0 was used as a semiflexible docking tool [29, 30]. The software was installed in a windows-based computer system with the following features: installed memory: 32GB; processor: Intel Core i7; system type: 64-bit. Twenty-six residues were observed inside the active site of the receptor, including Tyr36, Ile48, Ala52, Tyr53, Lys54, Val56, Ser57, Tyr64, Ala69, Lys71, Ile73, Tyr81, Arg84, Thr85, Gln105, Asp106, Leu107, Lys114, Asp123, Met125, Asp128, Lys131, Cys166, Asp167, Leu173, and Asp184. To avoid missing any atoms related to the residues cooperated in the MAPK3 active site, the grid box was set to 84, 60, and 70, pointing in *x*, *y*, and *z* coordinates, respectively, with a spacing of 0.375 Å. The grid box centers were 33.335 Å, 55.015 Å, and 49.3 Å for *X*, *Y*, and *Y* directions, respectively. Fifty conformations were constructed for each flavonoid using the Lamarckian genetic algorithm [31] and ranked based on their estimated binding energies.

### 2.3. Cross-Validation Study

The most potent MAPK3 inhibitors achieved from the AutoDock tool were selected for cross-validation study. In this regard, the Schrödinger Maestro docking tool version 10.2 was used to calculate the docking scores [32, 33]. The lowest dock score (Glide score) was assigned as the best-docked model for each component. Furthermore, the prime MM-GBSA approach was utilized to indicate the relative binding energies [34].

### 2.4. Molecular Dynamics

MD was executed in 100 ns (1,00,000 ps) simulations by Discovery Studio Client software version 16.1.0.15350 to evaluate the stability of the docked poses between the top-ranked flavonoids, based on the AutoDock tool and Schrodinger Maestro docking software and MAPK3 active site. Advanced settings for MD simulation are mentioned in our previous report [28]. Furthermore, the root mean square fluctuation (RMSF) of MAPK3 residues and the time evolution of root mean square deviation (RMSD) of the receptor backbone atoms complexed with the top-ranked flavonoids were analyzed. BIOVIA Discovery Studio Visualizer 19.1.0.18287 was used to unravel interactions between top-ranked flavonoids and residues inside the MAPK3 active site and to illustrate two- and three-dimensional views of their docked poses.

### 2.5. Pharmacokinetic and Toxicology Assessment

The SwissADME online web server, available at https://www.swissadme.ch/ [35], evaluated the selected flavonoids' absorption, distribution, metabolism, and excretion (ADME). Furthermore, the ligands' Lethal Dose 50 (LD50) was predicted using the ProTox-IIweb server available from https://tox.charite.de/protox_II/ [36].

## 3. Results

### 3.1. Binding Affinity Assessment Using AutoDock

**Figure 1:**
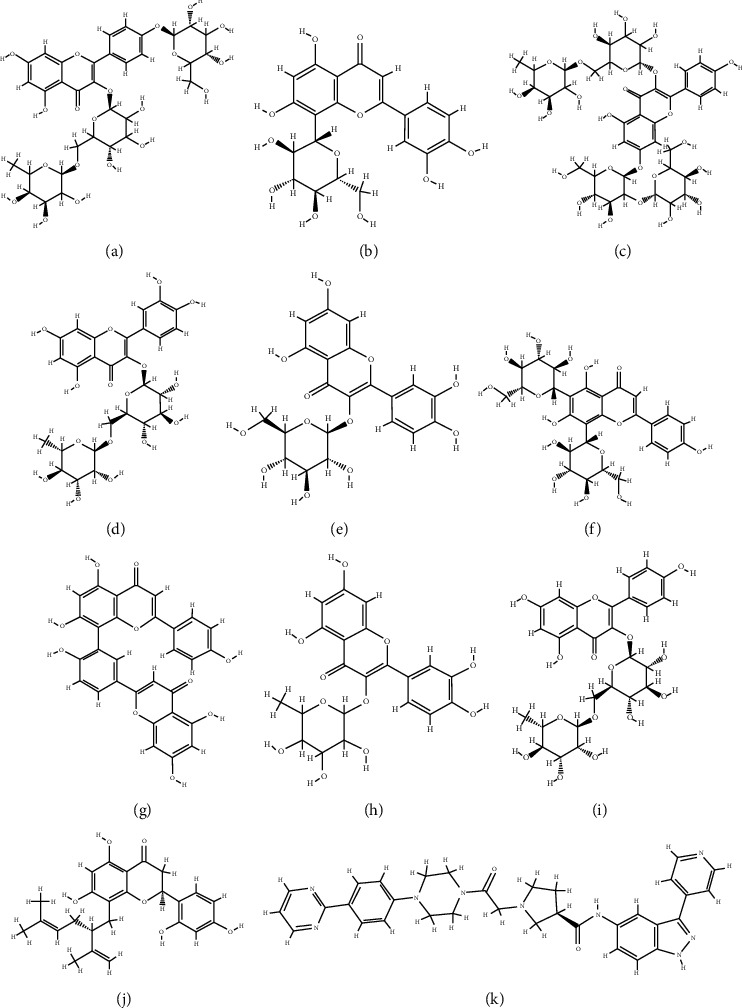
Chemical structures of (a) kaempferol 3-rutinoside-4′-glucoside, (b) orientin, (c) kaempferol 3-rutinoside-7-sophoroside, (d) rutin, (e) isoquercitrin G, (f) vicenin-2, (g) amentoflavone, (h) quercetin-3-rhamnoside, (i) nicotiflorin, (j) sophoraflavanone G, and (k) the standard drug (purvalanol).

**Figure 2:**
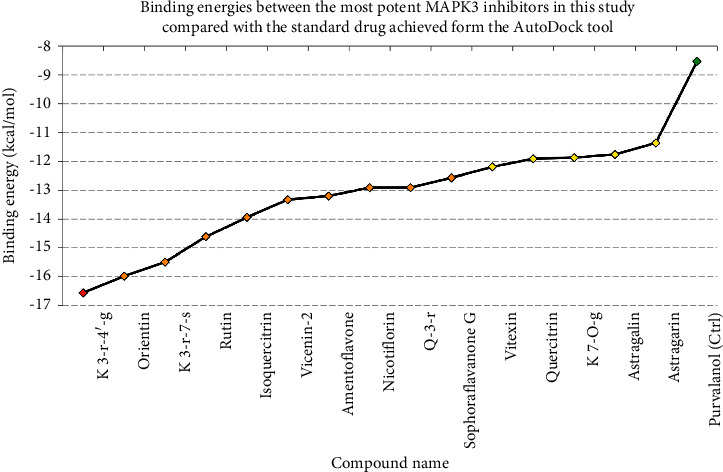
Estimated binding energies between these study's most potent MAPK3 inhibitors compared to the standard drug (purvalanol) achieved from the AutoDock tool. The *x*-axis illustrates the compound's names, and the *y*-axis shows their estimated binding energies (kcal/mol). The green diamond presents the purvalanol, while yellow, orange, and red spots represent flavonoids with inhibition constant values at nanomolar, picomolar, and femtomolar scales, respectively. MAPK3, mitogen-activated protein kinase 3.

According to the virtual screening analysis achieved by AutoDock 4.0, four and 32 compounds demonstrated Ki values at the micromolar (uM) and nanomolar (nM) scales, respectively. Besides, it was estimated that nine compounds, including orientin, kaempferol 3-rutinoside-7-sophoroside, rutin, isoquercitrin, vicenin-2, amentoflavone, quercetin-3-rhamnoside, nicotiflorin, and sophoraflavanone G, could potentially bind to the MAPK3 active site at the picomolar (pM) scale. Also, a salient binding affinity was observed between kaempferol 3-rutinoside-4′-glucoside (PubChem ID: 44258844) and MAPK3 catalytic site with the *K*i and Δ*G*_binding_ values of 731.68 femtomolar (fM) and −16.65 kcal/mol, respectively. Therefore, the present study calculated the *K*i value for ten compounds at either pM or fM concentrations. These flavonoids were considered top-ranked MAPK3 inhibitors among the studied flavonoids based on the AutoDock tool.  Figure 1 demonstrates the chemical structures of these flavonoids and purvalanol. The Δ*G*_binding_ value between purvalanol and MAPK3 active site was estimated as −8.53 kcal/mol. Accordingly, 39 flavonoids demonstrated a higher binding affinity to the MAPK3 catalytic site than the positive control inhibitor.  Figure 2 presents Δ*G*_binding_ values between top-ranked flavonoids achieved from the AutoDock tool, the standard drug, and MAPK3 active site. The estimated binding energies and *K*i values for 46 flavonoids and the control inhibitor in this study are presented in Table 1. Furthermore, the details of energies between top-ranked inhibitors and MAPK3 catalytic domain are shown in Table 2.

### 3.2. Cross-Validation Study Using Schrödinger Maestro Docking Tool

Cross-validation analysis was performed for kaempferol 3-rutinoside-4′-glucoside, orientin, kaempferol 3-rutinoside-7-sophoroside, rutin, isoquercitrin, vicenin-2, amentoflavone, quercetin-3-rhamnoside, nicotiflorin, sophoraflavanone G, and purvalanol. According to the results achieved from the Schrödinger Maestro docking tool, four metabolites, including kaempferol 3-rutinoside-4′-glucoside, kaempferol 3-rutinoside-7-sophoroside, rutin, and vicenin-2, demonstrated docking scores <−10 kcal/mol. These components assigned the most potent MAPK3 inhibitors based on the AutoDock 4.0 and Schrödinger Maestro docking tool version 10.2. Therefore, MD was executed to evaluate the strength of their docked poses in 100 ns computer simulation.  Table 3 presents the docking scores and MM-GBSA results calculated by the Schrödinger Maestro docking tool. The prime MM-GBSA analysis calculates the Δ*G*_binding_value between ligands and macromolecules.

### 3.3. Stability of the Docked Poses

**Figure 3:**
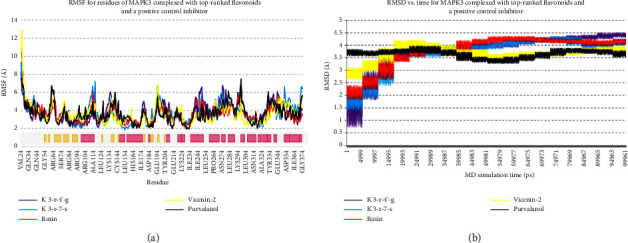
(a) RMSF and (b) RMSD for backbone atoms of MAPK3 complexed with top-ranked metabolites in this study and the standard drug. The secondary structure of MAPK3 was obtained from the PDB database; yellow and pink colors show beta-strand and *α*-helix secondary structures, respectively. MAPK3, mitogen-activated protein kinase 3; RMSD, root-mean-square deviations; RMSF, root mean square fluctuation.

**Figure 4:**
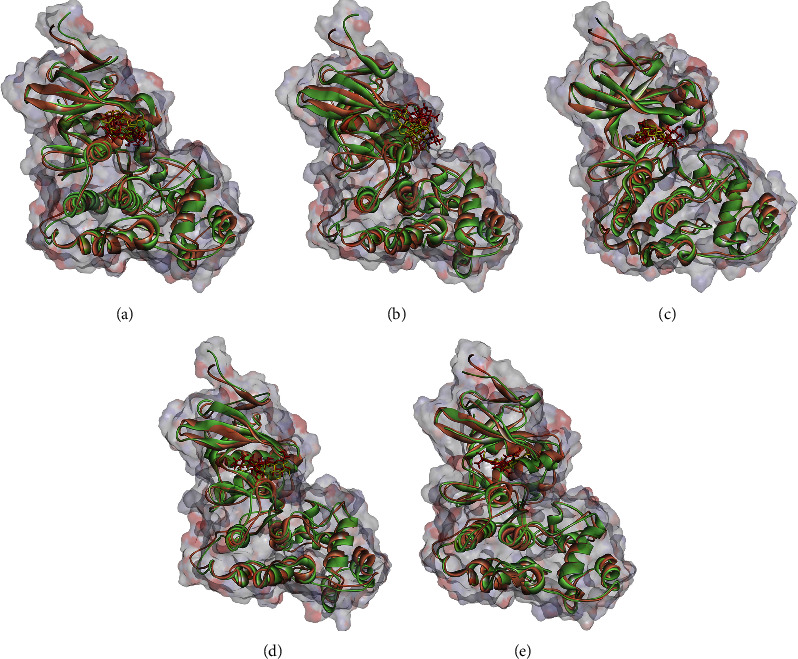
Superimposed structures of MAPK3 complexed with (a) kaempferol 3-rutinoside-4′-glucoside, (b) kaempferol 3-rutinoside-7-sophoroside, (c) rutin, (d) vicenin-2, and (e) pulvalanol before and after MD simulation analysis. Brown and green colors illustrate proteins before and after 100 ns MD simulations. Yellow and red compounds demonstrate ligands before and after MD analysis, respectively. MAPK3, mitogen-activated protein kinase 3.

Regarding MD analysis, the docked poses between kaempferol 3-rutinoside-4′-glucoside, kaempferol 3-rutinoside-7-sophoroside, rutin, vicenin-2, and MAPK3 active site were stable after ∼45 ns computer simulations.  Figure 3 demonstrates RMSF and RMSD for MAPK3 backbone atoms complexed with top-ranked flavonoids in this study and the standard drug.  Figure 4 illustrates the superimposed structures of top-ranked complexes before and after MD simulations.

**Figure 5:**
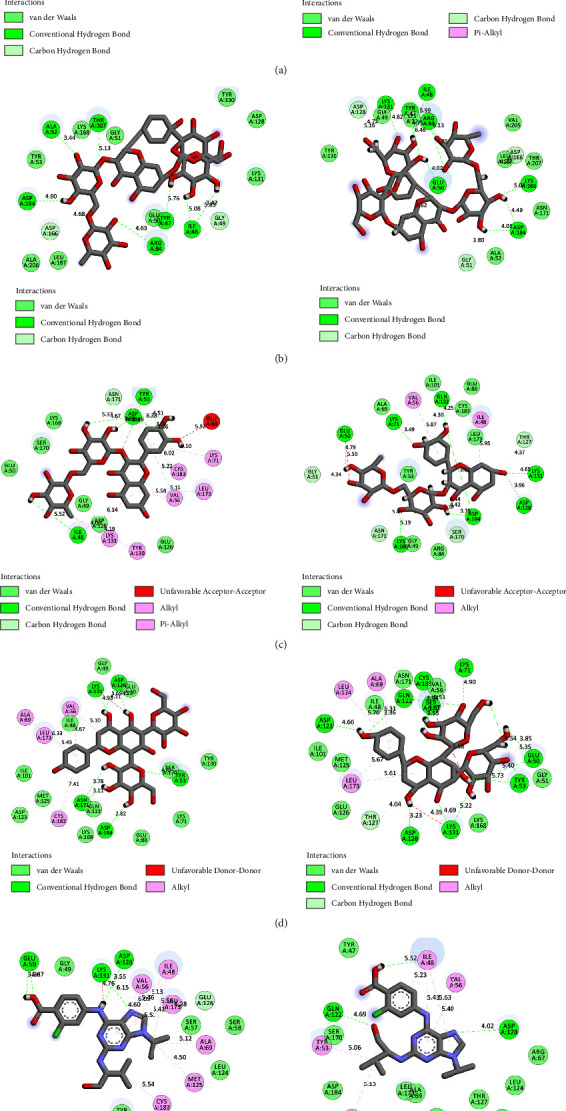
Interactions between amino acids inside the MAPK3 active site and (a) kaempferol 3-rutinoside-4′-glucoside, (b) kaempferol 3-rutinoside-7-sophoroside, (c) rutin, (d) vicenin-2, and (e) pulvalanol before (left images) and after (right images) MD simulations. MAPK3, mitogen-activated protein kinase 3; MD, molecular dynamics.

BIOVIA Discovery Studio Visualizer 19.1.0.18287 unraveled hydrogen bonds and hydrophobic interactions between top-ranked flavonoids (based on the AutoDock tool and Schrodinger Maestro docking software), purvalanol, and the enzyme's active site (Figure 5 and Table 4). The number of H bonds was increased after 100 ns computer simulations for kaempferol 3-rutinoside-4′-glucoside, kaempferol 3-rutinoside-7-sophoroside, rutin, and vicenin-2.

### 3.4. ADMET Assessment

SwissADME provides valuable information related to the pharmacokinetic features of compounds. The following ADME was predicted for 46 flavonoids studies in the present study: gastrointestinal (GI) and blood–brain barrier (BBB) permeability, P-gp (P-glycoprotein) substrate, cytochrome P-450 inhibition, and skin permeation coefficient (kp). Rutin and vicenin-2 revealed more appropriate ADME among top-ranked flavonoids. Besides, none of the compounds demonstrated considerable toxicity.  Table 5 lists the results of ADME and the toxicity of the compounds.

## 4. Discussion

MAPK3 is a serine/threonine kinase involved in the phosphorylation and translocation of several cytosolic proteins into the nucleus, leading to the dysregulation of several vital pathways and biological processes associated with apoptosis and cell proliferation [37]. Elevated expression and/or activity of MAPK3 is linked to the onset, development, drug resistance, and metastasis of various carcinomas such as ovarian cancer [38], glioma [39], lung cancer [40], and breast cancer [41]. Therefore, the present study executed a computational drug discovery approach to identify potential MAPK3 inhibitors from natural flavonoids, which have widely exhibited anticanter effects [42].

According to the AutoDock results, kaempferol 3-rutinoside-4′-glucoside demonstrated the highest binding affinity to the MAPK3 active site (Δ*G*_binding_ = −16.56 kcal/mol; *K*i = 731.68 fM) followed by orientin, kaempferol 3-rutinoside-7-sophoroside, rutin, isoquercitrin, vicenin-2, amentoflavone, nicotiflorin, quercetin-3-rhamnoside, and sophoraflavanone G. The cross-validation study also confirmed the high affinity of binding between kaempferol 3-rutinoside-4′-glucoside, kaempferol 3-rutinoside-7-sophoroside, rutin, vicenin-2, and MAPK3 active site. Therefore, these metabolites were considered the most potent MAPK3 inhibitors from the studied flavonoids.

Kaempferol 3-rutinoside-4′-glucoside is isolated from the fruits of Lyciumruthenicum Murr [43] and the leaves of Agave sisalana Perrine ex Engelm [44]. This compound demonstrated six H-bonds with Ile48, Glu50, Ala52, Lys131, Ser170, and Asp184 within the MAPK3 catalytic site before MD simulation. This flavonoid also exhibited 15 hydrogen and one hydrophobic interaction with Ala52, Tyr53, Ile48, Arg84, Asp128, Asp166, Lys168, Ser170, and Asn171, after 100 ns MD simulation. In addition, salient binding affinities were estimated between kaempferol 3-rutinoside-7-sophoroside, kaempferol 7-O-glucoside, and MAPK3 active site with the Δ*G*_binding_ value of −15.49 and −11.87 kcal/mol, respectively.

Kaempferol 3-rutinoside-7-sophoroside formed five hydrogen bonds with the Gly49, Ala52, Arg84, Asp166, and Asp184 located in the MAPK3 active site before MD simulation. This compound formed ten H bonds with the Tyr47, Glu50, Gly51, Arg84, Asp128, Lys131, Asp166, and Asp184, after 100 ns MD simulations. Besides, kaempferol showed a lower binding affinity to the MAPK3 catalytic site compared to that of its glycosylated forms with a Δ*G*_binding_ value of −8.5 kcal/mol, indicating a positive correlation between binding sugar moieties to the rings A, B, and C and enhancing binding affinity between kaempferol and MAPK3 active site. According to a previous report [14], kaempferol exhibited a considerable binding affinity to the matrix metalloproteinase 8 (MMP8) catalytic domain with a Δ*G*_binding_ value of −10.88 kcal/mol. Due to the critical role of MMP8 [45] and MAPK3 in cancer development and metastasis, kaempferol and its glycosylated forms could be considered drug candidates for cancer therapy.

Kaempferol is a yellow flavonoid [46] mainly found in apples, tomatoes, grapes, pine, green tea, and angelica [47]. Antioxidant and anti-inflammatory properties of kaempferol [46] may lead to therapeutic effects in various cancers by regulating cell cycle, apoptosis, metastasis, and angiogenesis [48]. Fouzder et al. [49] demonstrated that kaempferol diminished Nrf2 at mRNA and protein levels in nonsmall cell lung cancer cells, leading to the downregulation of Nrf2 downstream genes, including GST, AKR1C1, HO1, and NQO1, resulting in cancer cells sensitive to apoptosis. Pan et al. [50] executed an experimental study to examine the effects of kaempferol on rheumatoid arthritis (RA) *in vitro* and *in vivo*. The authors examined the cell migration and invasion using scratch assays and the Boyden chamber approaches, respectively. The cytoskeletal reorganization of RA fibroblast-like synoviocytes was tested using immunofluorescence staining. Real-time PCR and western blotting assays were used to examine the MMP expression levels. Pan et al. [50] reported that kaempferol diminished cell migration and invasion by downregulating the MAPK pathway, reduced the MMP expression, and inhibited the actin reorganization in RA FLSs. Huang et al. [51] demonstrated that kaempferol significantly inhibited the expression of several genes mediating inflammatory response, including interleukin (IL)-1*β*, cyclooxygenase-2, and nitric oxide synthase. Furthermore, kaempferol diminished collagen II degradations by inhibiting MMP1, MMP3, and MMP13 expression. In addition, kaempferol downregulated the p38 MAPK pathway. Taken together, Huang et al. [51] introduced kaempferol as a compound with therapeutic effects in osteoarthritis.

Orientin was this study's second leading potential MAPK3 inhibitor with the Δ*G*_binding_ and *K*i values of −15.98 kcal/mol and 1.92 pM, respectively. This flavonoid exhibited seven hydrogen and two hydrophobic interactions with the Gly49, Tyr53, Glu50, Val56, Asp128, Ser170, and Asn171 inside the MAPK3 catalytic site. Khamverdi et al. [52] also reported a considerable binding affinity between orientin and another protein kinase named glycogen synthase kinase 3 beta (GSK3B) with the criteria of Δ*G*_binding_ and *K*i values of −9.43 kcal/mol and 123.19 nM, respectively. Moreover, orientin has demonstrated a high affinity of binding to the MMP8 (Δ*G*_binding_ = −10.56 kcal/mol) [14] and MMP13 (Δ*G*_binding_ = −10.5 kcal/mol) [53] catalytic sites. The role of MMP13 in cancer progression [54] and metastasis [55] is evident. Therefore, orientin could be assigned as a potential drug candidate for cancer treatment with inhibitory effects against proteins involved in cancer onset, development, and metastasis.

Orientin is predominantly found in dayflower, millet, passion fruit, and pigeon pea leaves [56]. Several pharmacological features have been reported for orientin, such as antioxidant, anti-inflammatory, antimicrobial, and radio-protective effects [57]. Kim et al. [58] demonstrated that orientin inhibited the invasive behavior of breast cancer cells via downregulating MMP9 and interleukin (IL-8) expression. Furthermore, Tian et al. [59] reported that orientin reduced cell proliferation and enhanced the apoptosis process in T24 human transitional cell bladder carcinoma cells in vitro by inhibiting nuclear factor-kappaB (NF-*κ*B).

Wu et al. [60] demonstrated that mulberry ethanol extract (MBE) and rutin (the most abundant phenolic component in MBE) significantly reduced the expression of several genes mediating the MAPK pathway, including ERK, JNK, p38, and caspase-3, leading to reduced oxidative stress in gastric mucosal epithelial cells. This was done using the quantitative PCR method. Rutin demonstrated five hydrogen and nine hydrophobic interactions with the Ile48, Tyr53, Lys71, Tyr130, Lys131, Val56, Leu173, Cys183, and Asp184 before MD simulation. Besides, rutin exhibited 12 hydrogen and two hydrophobic interactions with the Ile48, Glu50, Gly51, Val56, Lys71, Glu122, Thr127, Asp128, Lys131, Ser170, Asn171, and Asp184 within the MAPK3 active site after 100 ns MD simulations.

Chen et al. [61] reported that vicenin-3 considerably reduced the expression levels of nitric oxide, prostaglandin E2, MMP1, MMP3, MMP13, and A disintegrin-like and metalloproteinase with thrombospondin motifs (ADAMTS). Moreover, vicenin-3 inhibited the p38 MAPK signaling pathway; the results were similar to that of SB203580 (a well-known p38 MAPK inhibitor). The present study evaluated the binding affinity between vicenin-2 and MAPK3 active site. Vicenin-2 showed seven hydrogen and five hydrophobic interactions with the Tyr53, Val56, Ala69, Ala128, Lys131, Asn171, Leu173, Cys183, and Asp184 before MD analysis. Furthermore, this compound illustrated ten hydrogen and four hydrophobic interactions with the Glu50, Lys71, Gln122, Asp123, Thr127, Asp128, Lys131, Ser170, and Cys183 after 100 ns computer simulations.

By analyzing the binding affinities between top-ranked MAPK3 inhibitors achieved from the AutoDock tool in this study and comparing the results with their corresponding structures, the following notes are suggested:

1. By comparing the results of kaempferol with its glycosylated forms, it might be suggested that binding a sugar moiety (or sugar moieties) to the basic structure of flavonoids elevates the binding affinity of the compound to MAPK3
2. By analyzing the Δ*G*_binding_ values between kaempferol 3-rutinoside-4′-glucoside, kaempferol 3-rutinoside-7-sophoroside, rutin, vicenin-2, and MAPK3 catalytic site, it could be hypothesized that binding a disaccharide to the ring C or two monosaccharides to the ring A considerably elevates the binding affinity of the compound to the MAPK3

## 5. Conclusion

In conclusion, the binding affinity of 46 flavonoids to the MAPK3 catalytic domain was estimated using the AutoDock tool. Thirty-nine flavonoids exhibited a higher binding affinity to the MAPK3 active site than the standard drug. A cross-validation study was executed on top-ranked flavonoids based on the AutoDock software. Kaempferol 3-rutinoside-4′-glucoside, kaempferol 3-rutinoside-7-sophoroside, rutin, and vicenin-2 exhibited excellent binding affinity using AutoDock 4.0 and Schrödinger Maestro docking tool. Also, these flavonoids' docked poses seemed stable after ∼45 ns MD simulations. The present study suggests kaempferol 3-rutinoside-4′-glucoside, kaempferol 3-rutinoside-7-sophoroside, rutin, and vicenin-2 as potent inhibitors of MAPK3. These findings may be helpful in the treatment of various cancers. However, *in vitro*, *in vivo*, and clinical trial studies are needed.

## Figures and Tables

**Table 1:** Binding energy and inhibition constant values for 46 flavonoids and a positive control inhibitor docked to the MAPK3 active site, achieved from the AutoDock tool.

PubChem ID	Ligand name	Inhibition constant (*k*i)	Binding energy (kcal/mol)
44258844	Kaempferol 3-rutinoside-4′-glucoside	731.68 fM	−16.56
5281675	Orientin	1.92 pM	−15.98
44258853	Kaempferol 3-rutinoside-7-sophoroside	4.41 pM	−15.49
5280805	Rutin	19.54 pM	−14.61
5280804	Isoquercitrin	60.05 pM	−13.94
442664	Vicenin-2	168.28 pM	−13.33
5281600	Amentoflavone	212.47 pM	−13.20
5318767	Nicotiflorin	343.03 pM	−12.91
5353915	Quercetin-3-rhamnoside	343 pM	−12.91
72936	Sophoraflavanone G	608.24 pM	−12.57
5280441	Vitexin	1.16 nM	−12.19
5280459	Quercitrin	1.86 nM	−11.91
10095180	Kaempferol 7-O-glucoside	1.98 nM	−11.87
5282102	Astragalin	2.39 nM	−11.76
9911508	Astragarin	4.69 nM	−11.36
5281672	Myricetin	20.66 nM	−10.48
439533	Taxifolin	25.43 nM	−10.36
5280704	Apigenin-7-glucoside	27.61 nM	−10.31
5280343	Quercetin	32.01 nM	−10.22
5316673	Afzelin	36.07 nM	−10.15
5280637	Cynaroside	39.83 nM	−10.10
5280681	3-O-methylquercetin	41.41 nM	−10.07
1203	Epicatechin	50.71 nM	−9.95
471	Dihydroquercetin	53.59 nM	−9.92
5280544	Herbacetin	62.60 nM	−9.83
5280445	Luteolin	65.25 nM	−9.80
5281654	Isorhamnetin	85.22 nM	−9.64
5281614	Fisetin	91.83 nM	−9.60
9064	Catechin	152.68 nM	−9.30
638278	Isoliquiritigenin	177.83 nM	−9.21
5318998	Licochalcone A	225.53 nM	−9.07
14309735	Xanthogalenol	280.13 nM	−8.94
5281670	Morin	298.46 nM	−8.90
629440	Hemileiocarpin	319.53 nM	−8.86
443639	Epiafzelechin	326.71 nM	−8.85
5281612	Diosmetin	385.31 nM	−8.75
5280443	Apigenin	433.98 nM	−8.68
124052	Glabridin	455.60 nM	−8.65
5317435	Fustin	480.39 nM	−8.62
5280863	Kaempferol	590.49 nM	−8.50
5281607	Chrysin	732.28 nM	−8.37
72281	Hesperetin	996.15 nM	−8.19
25201019	Ponciretin	1.01 *μ*M	−8.18
639665	XanthohuMol	3.20 *μ*M	−7.50
5280378	Formononetin	4.06 *μ*M	−7.36
10680	Flavone	1.74 *μ*M	−7.17
448991	Purvalanol (ctrl)	559.25 nM	−8.53

MAPK3, mitogen-activated protein kinase 3; ctrl, control.

**Table 2:** Details of energies between top-ranked flavonoids, positive control inhibitor, and MAPK3 catalytic site, achieved from the AutoDock tool.

Ligand name	Final intermolecular energy (kcal/mol)	Final total internal energy (kcal/mol)	Torsional free energy (kcal/mol)	Unbound system's energy (kcal/mol)	Estimated binding energy (kcal/mol)
Kaempferol 3-rutinoside-4′-glucoside	−9.04	−16.62	5.97	−3.14	−16.56
Orientin	−9.61	−10.86	3.58	−0.91	−15.98
Kaempferol 3-rutinoside-7-sophoroside	−6.54	−21.39	7.46	−4.98	−15.49
Rutin	−10.12	−12.91	5.07	−3.35	−14.61
Isoquercitrin	−10.82	−9.24	3.88	−2.24	−13.94
Vicenin-2	−11.49	−9.34	5.07	−2.43	−13.33
Amentoflavone	−9.89	−7.5	3.28	−0.92	−13.20
Quercetin-3-rhamnoside	−12.08	−6.52	3.28	−2.4	−12.91
Nicotiflorin	−11.48	−9.06	4.77	−2.86	−12.91
Sophoraflavanone G	−12.81	−3.1	2.68	−0.65	−12.57
Purvalanol (ctrl)	−9.26	−3.36	2.98	−1.1	−8.53

MAPK3, mitogen-activated protein kinase 3; ctrl, control.

**Table 3:** Schrödinger Maestro docking scores and relative binding free energies (kcal/mol) of top-ranked flavonoids (based on the AutoDock software) against MAPK3 active site (PDB ID: 4QTB; chain A).

Ligand name	Docking score (kcal/mol)	MMGBSA result (kcal/mol)
Kaempferol 3-rutinoside-4′-glucoside	−11.20	−42.65
Kaempferol 3-rutinoside-7-sophoroside	−11.03	−74.64
Vicenin-2	−10.32	−47.35
Rutin	−10.22	−25.88
Isoquercitrin	−9.25	−59.06
Nicotiflorin	−8.90	−44.55
Quercetin-3-rhamnoside	−7.34	−53.38
Sophoraflavanone G	−7.23	−55.43
Orientin	−7.09	−58.66
Amentoflavone	−7.04	−51.23
Purvalanol	−6.49	−47.01

**Table 4:** Interaction modes between top-ranked flavonoids and residues within the MAPK3 active site.

PubChem ID	Ligand name	Hydrogen bond (distance Å)	Hydrophobic interaction (distance Å)
44258844	Kaempferol 3-rutinoside-4′-glucoside (before MD)	Glu50 (2.97); Ile48 (4.64); Lys131 (4.99); Asp184 (4.58); Ala52 (3.91); Ser170 (4.47)	NA
44258844	Kaempferol 3-rutinoside-4′-glucoside (after MD)	Arg84 (4.39); Asp166 (4.15, 4.86); Asn171 (3.85); Lys168 (4.49, 4.67); Ser170 (3.90, 3.11, 3.27, 4.05); Asp128 (4.21, 3.57); Ile48 (4.33); Ala52 (3.80, 4.16)	Tyr53 (7.06)
44258853	Kaempferol 3-rutinoside-7-sophoroside (before MD)	Ala52 (3.44); Asp184 (4.80); Asp166 (4.68); Arg84 (4.63); Gly49 (3.42)	NA
44258853	Kaempferol 3-rutinoside-7-sophoroside (after MD)	Asp166 (4.77); Asp184 (4.49, 4.05); Gly51 (3.80); Asp128 (4.72); Lys131 (4.82); Tyr47 (4.75); Glu50 (4.02, 4.62); Arg84 (4.85)	NA
5280805	Rutin (before MD)	Asp184 (4.51, 3.06, 3.07, 3.89, 4.67)	Tyr53 (6.38); Cys183 (6.02); Lys71 (6.10); Val56 (5.22, 5.58); Leu173 (5.11); Ile48 (6.14); Tyr130 (4.19); Lys131 (4.00)
5280805	Rutin (after MD)	Thr127 (4.37); Lys131 (4.65); Asp128 (3.96); Asp184 (3.88, 3.75); Ser170 (4.42); Asn171 (3.40); Gly51 (4.34); Glu50 (4.79); Lys71 (3.49); Gln122 (4.30, 4.25)	Ile48 (5.95); Val56 (5.87)
442664	Vicenin-2 (before MD)	Asp128 (3.69, 4.23); Lys131 (4.93); Tyr53 (4.82); Asp184 (2.82); Asn171 (3.78, 3.11)	Val56 (5.10, 4.67); Leu173 (5.43); Ala69 (6.33); Cys183 (7.41)
442664	Vicenin-2 (after MD)	Glu50 (3.85); Lys131 (4.69); Asp128 (3.23); Thr127 (4.04); Asp123 (4.66); Gln122 (3.36); Cys183 (3.94); Lys71 (4.90); Ser170 (4.67, 3.55)	Ala69 (6.31); Leu124 (5.70); Leu173 (5.67, 5.61)
24866313	Purvalanol (before MD)	Glu50 (3.98, 4.07); Asp128 (3.55)	Val56 (6.09, 4.60); Ile48 (5.76, 5.13, 5.56); Leu173 (5.51, 5.41); Ala69 (5.12); Met125 (4.50); Cys183 (5.54)
24866313	Purvalanol (after MD)	Asp128 (4.02); Gln122 (4.69)	Val56 (5.40); Ile48 (5.23, 5.43, 5.63); Tyr53 (5.06); Cys183 (5.13)

MAPK3, mitogen-activated protein kinase 3; ctrl, control.

**Table 5:** Predicted ADMET of 46flavonoids in this study.

Ligand name	GI abs	BBB permeant	P-gpsubstrate	CYP1A2 inhibitor	CYP2C19 inhibitor	CYP2C9 inhibitor	CYP2D6 inhibitor	CYP3A4 inhibitor	Log kp	LD50 (mg/kg)
Kaempferol 3-rutinoside-4′-glucoside	Low	No	Yes	No	No	No	No	No	−12.85	5000
Orientin	Low	No	No	No	No	No	No	No	−9.14	1213
Kaempferol 3-rutinoside-7-sophoroside	Low	No	Yes	No	No	No	No	No	−14.98	5000
Rutin	Low	No	No	No	No	No	No	No	−11.66	5000
Isoquercitrin	Low	No	No	No	No	No	No	No	−9.6	1190
Vicenin-2	Low	No	No	No	No	No	No	No	−11.53	536
Amentoflavone	Low	No	No	No	No	No	No	No	−6.01	3919
Nicotiflorin	Low	No	No	No	No	No	No	No	−10.93	5000
Quercetin-3-rhamnoside	Low	No	Yes	No	No	No	No	No	−9.15	5000
Sophoraflavanone G	High	No	No	No	No	Yes	No	Yes	−4.79	2000
Vitexin	Low	No	No	No	No	No	No	No	−8.79	1190
Quercetin	High	No	No	Yes	No	No	Yes	Yes	−7.05	159
Kaempferol 7-O-glucoside	Low	No	No	No	No	No	No	No	−8.52	5000
Astragalin	Low	No	No	No	No	No	No	No	−8.52	5000
Astragarin	Low	No	No	No	No	No	No	No	−8.52	5000
Myricetin	Low	No	No	Yes	No	No	No	Yes	−7.4	159
Taxifolin	High	No	No	No	No	No	No	No	−7.48	159
Apigenin-7-glucoside	Low	No	Yes	No	No	No	No	No	−7.65	5000
Quercetin	High	No	No	Yes	No	No	Yes	Yes	−7.05	159
Afzelin	Low	No	No	No	No	No	No	No	−8.07	5000
Cynaroside	Low	No	Yes	No	No	No	No	No	−8	5000
3-O-methylquercetin	High	No	No	Yes	No	No	Yes	Yes	−6.31	5000
Epicatechin	High	No	Yes	No	No	No	No	No	−7.82	10000
Dihydroquercetin	High	No	No	No	No	No	No	No	−7.48	2000
Herbacetin	High	No	No	Yes	No	No	Yes	Yes	−6.6	3919
Luteolin	High	No	No	Yes	No	No	Yes	Yes	−6.25	3919
Isorhamnetin	High	No	No	Yes	No	No	Yes	Yes	−6.9	5000
Fisetin	High	No	No	Yes	No	No	Yes	Yes	−6.65	159
Catechine	High	No	Yes	No	No	No	No	No	−7.82	10000
Isoliquiritigenin	High	Yes	No	Yes	No	Yes	No	Yes	−5.61	1048
Licochalcone A	High	Yes	No	Yes	Yes	Yes	No	Yes	−4.89	1000
Xanthogalenol	High	No	No	Yes	No	Yes	No	Yes	−4.86	1000
Morin	High	No	No	Yes	No	No	Yes	Yes	−7.05	3919
Hemileiocarpin	High	Yes	Yes	Yes	Yes	Yes	Yes	Yes	−5.59	500
Epiafzelechin	High	No	Yes	No	No	No	No	No	−7.46	2500
Diosmetin	High	No	No	Yes	No	Yes	Yes	Yes	−5.93	3919
Apigenin	High	No	No	Yes	No	No	Yes	Yes	−5.8	2500
Glabridin	High	Yes	Yes	Yes	Yes	Yes	Yes	Yes	−5.52	500
Fustin	High	No	No	No	No	No	No	No	−7.44	2000
Kaempferol	High	No	No	Yes	No	No	Yes	Yes	−6.7	3919
Chrysin	High	Yes	No	Yes	No	No	Yes	Yes	−5.35	2500
Hesperetin	High	No	Yes	Yes	No	No	No	Yes	−6.3	2000
Ponciretin	High	Yes	Yes	Yes	Yes	No	No	Yes	−6.02	2000
Xanthohumol	High	No	No	Yes	No	Yes	No	Yes	−4.86	3800
Formononetin	High	Yes	No	Yes	No	No	Yes	Yes	−5.95	2500
Flavone	High	Yes	No	Yes	Yes	No	No	No	−5.13	2500
Purvalanol (standard drug)	High	No	No	No	No	Yes	No	Yes	−6.24	465

GI, gastrointestinal; abs, absorption; BBB, blood–brain barrier; P-gp, p-glycoprotein; CYP, cytochrome p-450; Kp, skin permeation coefficient; LD50, lethal dose 50%.

## Data Availability

The datasets used and/or analyzed during the current study are available from the corresponding author on reasonable request.

## Abbreviations

MAPK3: Mitogen-activated protein kinase 3
ERK1: Extracellular signal-regulated kinase 1
*K*i: Inhibition constant
RMSF: Root mean square fluctuation
RMSD: Root mean square deviation
MD: Molecular dynamics
*μ*M: Micromolar
nM: Nanomolar
pM: Picomolar
ns: Nanosecond.
